# Evaluation of support group interventions for children in troubled families: study protocol for a quasi-experimental control group study

**DOI:** 10.1186/1471-2458-14-76

**Published:** 2014-01-24

**Authors:** Annemi Skerfving, Fredrik Johansson, Tobias H Elgán

**Affiliations:** 1 FORUM, Department of Clinical Neuroscience, Stockholm Centre for Psychiatric Research and Education, Stockholm County Council Health Care Provision and Karolinska Institutet, Stockholm, Sweden; 2 STAD, Department of Clinical Neuroscience, Stockholm Centre for Psychiatric Research and Education, Stockholm County Council Health Care Provision and Karolinska Institutet, Stockholm, Sweden

**Keywords:** Child of impaired parents, Support groups, Controlled trial, Coping behaviour, Coping skills, Mental health, Self-perception, Self-esteem, Personal satisfaction

## Abstract

**Background:**

Support groups for children in troubled families are available in a majority of Swedish municipalities. They are used as a preventive effort for children in families with different parental problems such as addiction to alcohol/other drugs, mental illness, domestic violence, divorce situations, or even imprisonment. Children from families with these problems are a well-known at-risk group for various mental health and social problems. Support groups aim at strengthening children’s coping behaviour, to improve their mental health and to prevent a negative psycho-social development. To date, evaluations using a control-group study design are scarce. The aim of the current study is to evaluate the effects of support groups. This paper describes the design of an effectiveness study, initially intended as a randomized controlled trial, but instead is pursued as a quasi-experimental study using a non-randomized control group.

**Methods/design:**

The aim is to include 116 children, aged 7–13 years and one parent/another closely related adult, in the study. Participants are recruited *via* existing support groups in the Stockholm county district and are allocated either into an intervention group or a waiting list control group, representing care as usual. The assessment consists of questionnaires that are to be filled in at baseline and at four months following the baseline. Additionally, the intervention group completes a 12-month follow-up. The outcomes include the Strength and Difficulties Questionnaire (SDQ S11-16), the Kids Coping Scale, the “Ladder of life” which measures overall life satisfaction, and “Jag tycker jag är” (I think I am) which measures self-perception and self-esteem. The parents complete the SDQ P4-16 (parent-report version) and the Swedish scale “Familjeklimat” (Family Climate), which measures the emotional climate in the family.

**Discussion:**

There is a need for evaluating the effects of support groups targeted to children from troubled families. This quasi-experimental study therefore makes an important contribution to this novel field of research. In the article various problems related to pursuing a study with children at risk are discussed.

**Trial registration:**

ISRCTN52310507

## Background

Children who grow up in families with parental problems like for instance mental health or substance abuse problems, run an increased risk of developing a number of different physical and psychological health issues, as well as social problems (*e.g.*, [1-6]). Available figures indicate that this is widespread, affecting many children who experience one or more of these problems in their families. For instance, international estimates indicate that 12-39% of all children have parents with mental health problems [7-10]; 10-40% are affected by domestic violence [11-13]; 8-30% grow up with at least one problem drinking parent [14-17]; about 2% of US children have a parent in prison [18]. In Sweden, figures are similar, revealing that about 6% of all children aged 0–17 have at least one parent who has received inpatient psychiatric care [19], and approximately 20% have parents with alcohol problems [20,21], while about 30% of all children have divorced parents (http://www.scb.se) and approximately 2% have a parent in prison [22].

Support is available to these children *via* for instance the Child and Adolescent Psychiatric Care (BUP), the general school health care and the Social Services, who provide support through Family Centres (Familjecentraler), Contact Families (where children can spend weekends) [23] and in severe cases, foster care. Additionally, a relatively novel way to offer intervention programs to this target group is by the use of the Internet and there are currently a small number of internet-based controlled trials ongoing [24,25]. The most commonly offered intervention in Sweden to children in troubled families is support groups run by the Social Services and/or NGOs. Regardless of the underlying problem within the family, these support group interventions are all derived from the same fundamental psycho-educative manual-based intervention called Children are People Too (CAP) [26-28]. The support group interventions are aimed at strengthening children’s feeling of self-worth, their competencies and coping behaviour, and thereby to prevent a negative psycho-social development. Briefly, the groups provide information about the parent’s problems and children are able to share their experiences with other children during 8–15 weekly one and a half hour long meetings each addressing a special theme.

There are only a few estimates available reporting on the number of existing support groups in Sweden. Moreover, figures on how many children there are who attend support groups every year are even scarcer. According to a survey from 2009 by the Swedish National Institute of Public Health [29], support groups for children in families with parental substance abuse problems were available in 90% of all municipalities. For children affected by parental mental illness, the same figure was 73%, for children who have witnessed domestic violence the figure was 64% and 38% for children having a parent in prison. More recent surveys made by the Swedish Save the Children and the junior association of the Swedish IOGT-NTO (Junis) also show that the vast majority of municipalities provide support groups to children experiencing parental problems [30,31]. Yet, despite the fact that most municipalities do provide resources for support, only a small proportion of all children who live in troubled families attend this support. For instance, in an annual survey by Junis figures reveal that only 1-2% of all children who have parents with substance abuse problems attend support groups [31].

To our knowledge there are few controlled trials reported in the literature, measuring the effects of support groups with a theoretical basis in CAP. However, a few studies have been conducted using a quantitative approach, but without a control group. For instance, a Swedish effect study of 300 children aged 7–22 [26] was pursued without a control group, but using added value as the method of analyses [32,33]. Results revealed an improvement among the participants with regards to their mental health status, overall life satisfaction and hopefulness [26]. Results from another study using a small group of children who had attended a CAP-based support group after experiencing family violence, indicated an improved mental health status among the participating children [34]. Nonetheless, there is still a lack of evidence for CAP-based support groups and we have therefore designed a quasi-experimental controlled trial which evaluates the effectiveness of support group interventions targeted to children experiencing a wide variety of parental problems. Our hypothesis is that these support groups will render in positive effects among 7–13 year olds.

### Objective and research questions

The objective of this study is to evaluate the effectiveness of support groups provided to children who grow up in families with parental problems (*i.e.,* addiction to alcohol and/or other drugs, mental illness, domestic violence, divorce situation or imprisonment). Specific research questions concerns the children’s improvement in mental health, coping behaviour and quality of life, which includes overall life satisfaction and future hope, self-perception and self-esteem, and emotional climate and personal interaction.

## Methods/design

**Figure 1:**
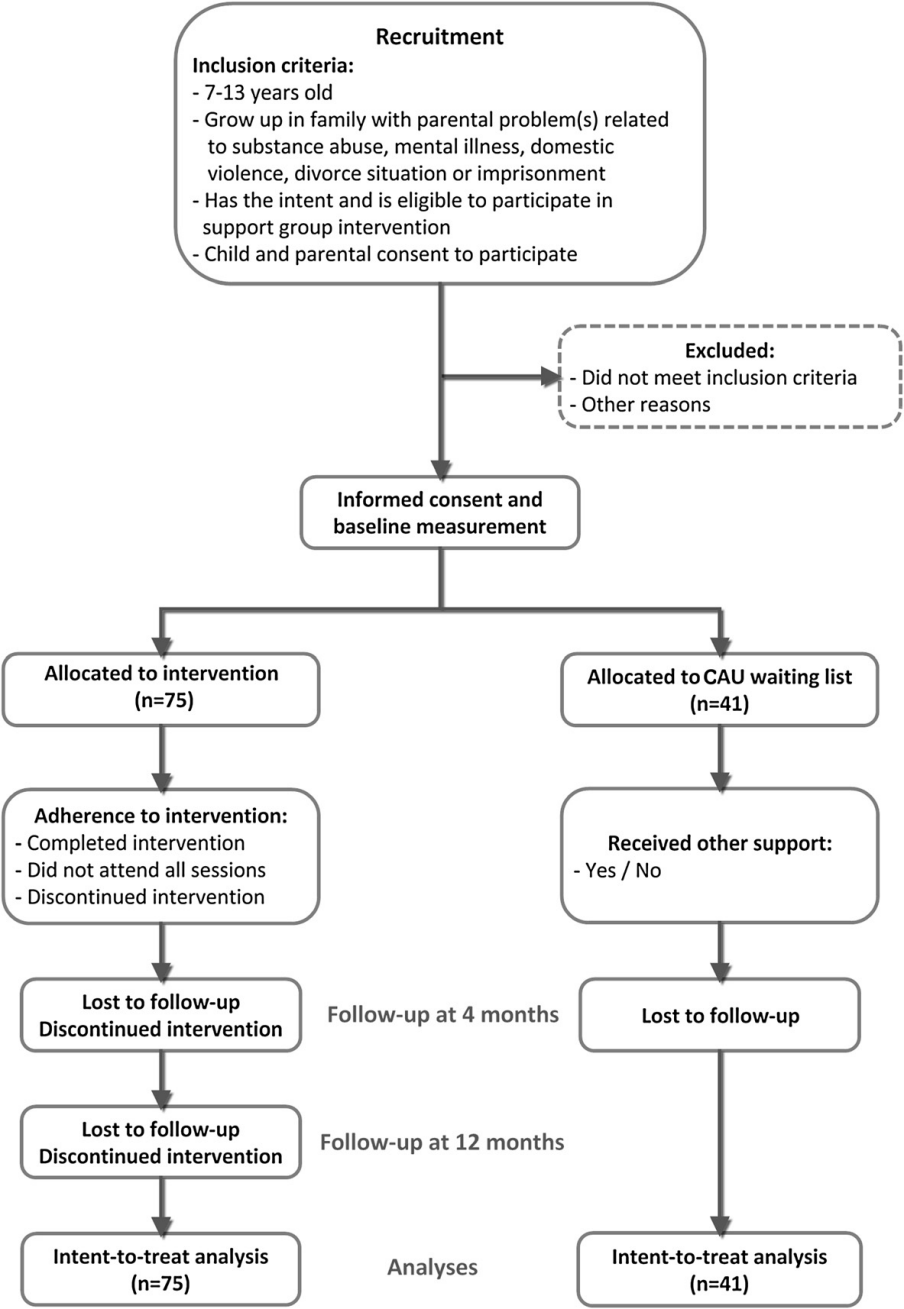
Flow chart diagram representing the design of the study.

This study was originally designed as a two-armed randomized controlled trial (RCT). However, after several months of unsuccessful recruitment efforts, the randomization protocol was abandoned and the study redesigned as a quasi-experimental control group study (Figure 1). The reason for abandoning the RCT-design was the unwillingness of support group therapists to randomize participants into the control condition. This was due to the fact that support group therapists did not consider it ethically sound to randomly allocate children, who actively had sought support (by themselves or by their parents), into the control condition.

### The target sample

The aim is to include at least 116 children, aged between 7 and 13 years at the point of enrolment, who intend and are eligible to participate in existing support group interventions targeted to children who grow up and live in families with at least one of the following parental problems: (1) addiction to alcohol and/or other drugs, (2) mental illness, (3) domestic violence, (4) divorce situation or (5) imprisonment. Participants will be excluded from the study if they do not meet the inclusion criteria, if they previously have participated in a support group intervention, or if they (or their parents) do not consent to participate.

### Recruitment

Recruitment has been ongoing since March 2010 *via* schools, the social services and various existing support groups for children having parents with any of the above mentioned problems. Additionally, there has been advertisments in free newspapers (Metro) which is locally distributed in the Stockholm and Uppsala region and the local newspaper in Stockholm (Mitt i). The catchment area of the study is the Stockholm county district, although a support group located in the Uppsala Region has been contacted and asked to contribute to the recruitment.

Staff at schools, adult mental health clinics and social services, along with support group therapists have been informed about the study and instructed on how to inform both children and their parent(s) about the study. To facilitate this work, pamphlets describing the study have been provided to those concerned. To participate in the study, parents or support group therapists need to visit a website which has been created specifically for this study (http://www.barngruppstudien.se), and apply for enrolment. Once signed up, the project manager sends out further information and a written informed consent to the parents/custodians and to the child, to be sent back to the project manager using a prepaid reply envelope. For the child to be included in the study, the written informed consent has to be signed by at least one parent/custodian. If only one parent signs the consent form, a passive parental consent procedure is adopted, which means that the other parent is informed and reminded of his/her right to say no.

### Assessment

Before being allocated to one of the two study conditions, participants have to complete the baseline measurement. All study participants also have to complete a follow-up assessment four months after the initial measurement. Additionally, the intervention group participants have to complete a second follow-up assessment 12 months after the initial measurement. This 12 month follow-up will only be available for the intervention group participants as the control group participants, for ethical reasons, will be offered the intervention after the four-month follow-up. All measurements are performed using paper-based questionnaires. The participants in the intervention group complete the initial baseline assessment during the first individual meeting, which precedes the first support group meeting. Baseline data from the interventions group is collected by the support group therapist responsible for the individual meeting, or by the project staff. Data from the four month follow-up assessments are collected by the project staff. For the 12 month follow-up (intervention group only), questionnaires are either distributed by regular mail (with telephone support available if needed) or in a meeting with the project staff (if requested by the child/parent). Data from the control group participants will be distributed and collected by the project staff in person or by using regular mail with telephone support if necessary. As a compensation for completing each assessment, children and parents are each given a gift card corresponding to approximately 22 or 11 Euro, respectively.

### Outcome measurements

#### Primary outcomes

To measure overall mental health, the Swedish version of a three-point likert scales, the Strength and Difficulties Questionnaire (SDQ; [35,36]) (SDQ-swe) is used. The SDQ-swe P4-16 version is to be filled out by the parent(s) of those children who are 7–10 year olds while the SDQ-swe S11-16 is a self-completion scale for those children who are 11–13 years old. The original SDQ and the Swedish version have been demonstrated to be reliable and valid among samples of 11–16 and 6–10 year olds, respectively [36,37].

Overall life satisfaction and future hope is measured by asking the children about their past, present- and future life rating, on a ten-point “Ladder of life” which represents life status from “worst” to “best” possible life imaginable [38-40]. The original version [38] was designed for adults and asked the respondents to consider a five-year perspective. A modified version asks children to rate their life satisfaction using a shorter time-frame of one year [39] and is used in this study for all children.

Self-perception and self-esteem is measured using the Swedish scale “Jag tycker jag är” [I think I am] [41] and is scored by the children themselves. This scale has previously been demonstrated to be reliable among Swedish university students [41,42].

Emotional climate and personal interaction within the family is measured using the Swedish scale “Familjeklimat” [Family Climate] (Hansson 1989) and is scored by the parents. This instrument consists of 85 adjectives which can be subdivided into four dimensions: “closeness” pertains to adjectives describing a positive climate with warmth, safety and harmony; “distance” comprise adjectives describing a negative atmosphere governed by coolness and rejection; “spontaneity” relates to adjectives describing the family’s emotional expressiveness in both positive and less positive terms; “chaos” pertains to adjectives that relates to a state of disorder within the family.

Coping behaviour is measured using three-point likert scales, the Kids Coping Scale (KCS; [43]), which has been translated into Swedish for the purpose of this study. This scale has demonstrated low to moderate levels of internal consistency and validity among a cross-sectional sample of 7–13 year olds [43] and is to be filled out by the children themselves.

### Additional outcomes

Adherence is measured using a self-constructed questionnaire, which has been used in a previous study by our research group [40], and contains 55 questions directed to support group therapists concerning the content of the support groups.

### Allocation

After completing the baseline assessment, participants are allocated to the intervention or the control group which represents care as usual (CAU). Due to the aforementioned reasons for abandoning the original randomization protocol, the vast majority of the control group participants are recruited from the support groups’ own waiting lists. Participants were informed about their group belonging by the project manager.

### The interventions

This study includes evaluating the effects of support groups in general that are aimed at children growing up in families with the above mentioned parental problems (*i.e.,* addiction to alcohol and/or other drugs, mental illness, domestic violence, divorce situation or imprisonment). Although these support groups are targeted at children with various underlying problems, they are all derived from the same fundamental manual-based intervention, Children are People Too (CAP) [26-28]. Depending on which programme/manual is being used, the design varies somewhat but generally consists of 8–15 group sessions (each between 90–120 minutes) discussing different aspects of parental problems in the family. Each session contains lectures related to the specific problem that the group aims at, and various games, role plays and practices related to family problem and coping behaviour [44,45]. Furthermore, every meeting focuses on a special theme and is structured in the same fashion, thereby making it recognisable from time to time to get children to feel more comfortable.

### The control

The control condition consists of a CAU waiting list. In Sweden, apart from support group interventions, usual care may involve support provided by for instance the social services (*e.g.,* contact families) and the school health care services, although it should be noted that this type of support is very uncommon. In severe cases children are referred to the Child and Adolescent Psychiatric Services.

### Sample size

This trial is designed to detect a medium or larger effect size corresponding to a standardized mean difference of Cohen’s d >0.5 [46]. Our hypothesis is directional in favour of the intervention. Based on differences between means, an *a priori* calculation of the estimated sample size, using the G*Power software [47] where an allocation ratio of 1.8 has been set, reveals that it is required that a minimum of 116 participants (75 in the intervention group and 41 in the control group) enrol in the trial (power = 0.80, α = 0.05, one tailed).

### Analyses

In addition to per protocol analysis, and if applicable, data will be analysed according to the intention-to-treat principle where all participants will be included irrespective of whether or not they have completed the intervention. Missing data will then be handled by multiple imputation using the Missing Value Analysis routine in the SPSS software (IBM SPSS Statistics 20, IBM Corporation).

The main analyses consist of comparing mean-values of outcome measurements between groups and within groups at the baseline and follow-up assessments. A follow-up at 12 months past the baseline is only conducted for the intervention group, precluding a between groups analysis at 12 months. Before analysis, all data will be merged and thus treated equally, irrespective of the nature of the support group (*i.e.,* parental problems related to for instance substance abuse, mental illness or domestic violence) that the child has participated in. Hence, the current design will not suffice analysis where data to a greater detail are differentiated based on the various support group interventions. Analyses include either parametric or non-parametric tests, depending on whether or not the various outcome data are normally distributed. The effects of the support group interventions will be estimated using Cohen’s convention of effect size [46].

### Ethics

This study has been approved by The Regional Ethical Review Board at the Karolinska Institutet (registration nr. 2010/5:4 and 2010/5:12).

## Discussion

This paper describes a quasi-experimental study of support group interventions for children who grow up in families with parental psycho-social problems. The various support groups included are all derived from the same manual-based CAP-intervention. The effectiveness of the groups will be evaluated using a controlled study design with two conditions: one group having access to the interventions and another group consisting of a waiting list control group. Originally, the study was designed as an RCT. However, after a few months of recruitment efforts it became clear that randomization of participants was not an option for the following reasons. First, many support group therapists expressed doubts about the RCT-design as they thought it was unethical to randomize children into the control condition. Additionally, once parents had made the decision to let their children participate in a group, many did not want their children to be allocated to a waiting list. This led us to conclude that if we retain the randomization protocol we run an imminent risk of not being able to pursue this study. Moreover, the difficulties in recruiting children at-risk into scientific studies, and the phenomenon of adult gatekeepers who restrain children’s participation [48,49], are well-known. Hence, at an early stage we abandoned the randomization protocol and instead use a more pragmatic approach, where the waiting list control group in fact consists of a “natural” waiting list on hold for entering a support group.

### Strengths and limitations

Support groups for children in troubled families based on the CAP-methodology, have gained a huge spread in Sweden as the vast majority of municipalities offer this as the principal intervention to this target group. However, to our knowledge there is no evidence for the effectiveness of these intervention programs. One strength of the present study is therefore that it attempts to investigate the effects of these support groups using a control-group design. Another strength is the fact that the study has the characteristics of an effectiveness trial, as it evaluates the effects of support groups already existing within the municipalities’ regular work. In this respect, it should also be mentioned that since the interventions under study are already implemented in the municipalities’ regular work, following this study, the dissemination work of the interventions will be minimal.

There are a number of possible limitations to this study. First, the design of the study is quasi-experimental as the original randomization protocol was abandoned. Hence, this study may be subjected to selection bias, as between-group baseline characteristics may differentiate. Further, since this study evaluates the effectiveness of support groups in general, all of which having a theoretical basis in CAP, children with various kinds of underlying problems will be included. Hence, there may be some systematic differences between the children which is based on their group belonging (*e.g.*, substance abusing parents, divorced parents etc.). Additionally, the study will not be stratified based on group belonging, which may have the consequence that children attending one type of support group may be overrepresented (*e.g.*, children having divorced parents may be overrepresented as this is more prevalent and less associated with stigma relative for instance having a parent in prison). The relatively short follow-up time period is a final limitation as between-group comparisons can only be made at the four-month follow-up. Thus, although the study will provide within-group comparisons after 12 months, this study will not generate any firmer evidence for long-term effects of support group interventions.

### Implications for practice

In Sweden, support groups for children in troubled families are the principal means of intervention offered to this target group. However, to date the number of papers describing effects of support group interventions for children in troubled families, which have their theoretical basis in CAP, are scarce and there is a request for evidence from both practitioners and policy-makers. In fact, the National Board of Health and Welfare in Sweden explicitly states that there is an ethical requirement that interventions that are commonly in use should be scientifically tested and at least be found not to cause any harm – it is not enough that individual social workers or other professionals believe that a given intervention is good. This study therefore makes an important and novel contribution to both the research literature and practice.

## Competing interests

The authors declare that they have no competing interests.

## Authors’ contributions

A.S. obtained funding, conceived, designed and coordinated the study and drafted the manuscript. F.J. performed the statistical analysis and contributed to the design of the study. T.H.E. contributed to the design of the study, statistical analysis and drafted the manuscript. All authors read and approved the final manuscript.

## Pre-publication history

The pre-publication history for this paper can be accessed here:

http://www.biomedcentral.com/1471-2458/14/76/prepub
